# The OSCAR-IB Consensus Criteria for Retinal OCT Quality Assessment

**DOI:** 10.1371/journal.pone.0034823

**Published:** 2012-04-19

**Authors:** Prejaas Tewarie, Lisanne Balk, Fiona Costello, Ari Green, Roland Martin, Sven Schippling, Axel Petzold

**Affiliations:** 1 MS Centre Amsterdam, VU University Medical Centre, Amsterdam, The Netherlands; 2 University of Calgary, Departments of Clinical Neurosciences and Surgery, Calgary, Alberta, Canada; 3 Multiple Sclerosis Center, Department of Neurology, University of California San Francisco, San Francisco, California, United States of America; 4 Institute for Neuroimmunology and Clinical Multiple Sclerosis Research (Inims), University Medical Center Hamburg Eppendorf, Hamburg, Germany; 5 UCL Institute of Neurology, London, United Kingdom; Institute Biomedical Research August Pi Sunyer (IDIBAPS) - Hospital Clinic of Barcelona, Spain

## Abstract

**Background:**

Retinal optical coherence tomography (OCT) is an imaging biomarker for neurodegeneration in multiple sclerosis (MS). In order to become validated as an outcome measure in multicenter studies, reliable quality control (QC) criteria with high inter-rater agreement are required.

**Methods/Principal Findings:**

A prospective multicentre study on developing consensus QC criteria for retinal OCT in MS: (1) a literature review on OCT QC criteria; (2) application of these QC criteria to a training set of 101 retinal OCT scans from patients with MS; (3) kappa statistics for inter-rater agreement; (4) identification reasons for inter-rater disagreement; (5) development of new consensus QC criteria; (6) testing of the new QC criteria on the training set and (7) prospective validation on a new set of 159 OCT scans from patients with MS. The inter-rater agreement for acceptable scans among OCT readers (n = 3) was moderate (kappa 0·45) based on the non-validated QC criteria which were entirely based on the ophthalmological literature. A new set of QC criteria was developed based on recognition of: (O) obvious problems, (S) poor signal strength, (C) centration of scan, (A) algorithm failure, (R) retinal pathology other than MS related, (I) illumination and (B) beam placement. Adhering to these OSCAR-IB QC criteria increased the inter-rater agreement to kappa from moderate to substantial (0.61 training set and 0.61 prospective validation).

**Conclusions:**

This study presents the first validated consensus QC criteria for retinal OCT reading in MS. The high inter-rater agreement suggests the OSCAR-IB QC criteria to be considered in the context of multicentre studies and trials in MS.

## Introduction

A consistent finding in patients with multiple sclerosis (MS) and MS related optic neuritis (MSON) is thinning of the retinal nerve fibre layer (RNFL), assessed by optical coherence tomography (OCT) [1], [2]. Loss of RNFL thickness is correlated with a number of clinical scales and brain imaging evidence for atrophy, suggesting a pathological link to neurodegeneration. This data is consistent with post–mortem evidence for neurodegeneration in about 80% of eyes from patients with multiple sclerosis [3]. Consequently, it has been proposed to investigate the value of OCT measures of retinal atrophy as a potential secondary outcome in neuroprotective treatment trials in MS [4], [5].

As one pre-requisite for such a trial it will be necessary to validate the accuracy of RNFL thickness assessment in a multi–centre setting. An important step towards this goal is the assessment of OCT scans by well trained readers in a reading center. This assessment judges on the quality of an OCT scan whether or not it can be included into a study. A review of the ophthalmological literature shows that poor scan quality is frequently caused by boundary line errors, poor signal strength or de-centration of the ring scan at the optic nerve head (ONH) [6]. There are, however, no consensus quality control criteria for RNFL assessment in patients with MS and MSON.

This study aimed to develop for the first time reliable and transparent consensus criteria for the quality assessment of retinal OCT scans in MS and MSON for application in the context of multi–centre studies.

## Methods

Retinal images were obtained using a Spectral Domain (SD)–OCT device (Heidelberg Spectralis, Software version 1.1.6.3) with the eye tracking function enabled. The OCT scans were recorded by qualified OCT operators at all centers: Amsterdam, Calgary, Hamburg and University of California San Francisco. In all patients a ring scan (diameter 12° or 2.4 cm) at the optic nerve head (ONH) was recorded. Given the purpose of this study, all scans, independent of either eye, were analysed together.

All scans were anonymised and uploaded to an OCT reading center (Amsterdam). The OCT scans were then rated independently by three trained raters (PT, LB, AP) in Amsterdam. The first set of scans (101 eyes from 51 patients, Hamburg) was rated in random order, based on published criteria [6]. Scans failing on these criteria were rated as “reject”. Scans were rejected following published recommendations on: decentration, poor scan quality, boundary line errors or algorithm failures [6].

Kappa statistics for multiple raters were calculated to assess the inter–rater agreement using the magree macro in SAS software (V9.2) [7]. The level of agreement was rated as slight (0–0.2), fair (0.2–0.4), moderate (0.4–0.6), substantial (0.6–0.8) or almost perfect (0·8–1) [8].

Next, the results were analysed to identify the main sources of disagreement. Based on this consensus a new set of criteria was summarised and tested on the training set of OCT scans from the Hamburg site in random order to check whether the inter-rater agreement could be improved. Finally, the consensus criteria were validated prospectively on a new set of 159 OCT scans from dedicated MS centers in Amsterdam, Calgary and UCSF. Again kappa statistics were used for data analyses.

### Ethics Statement

All participants signed informed consents and in all centres this study was approved by the Medical Ethics Comittee and the Commission for Scientific Research at the VU University Medical center.

## Results

The inter–rater agreement based on published quality control criteria was moderate (kappa 0.45) for the 101 OCT scans from Hamburg. The main sources of disagreement were the overal scan quality (26%) and ring scan de-centration (22%).

The revised consensus quality control criteria are summarised in Table 1. Each of the seven criteria is identifiable by the first letter, together giving the acronym “OSCAR IB” [(O) = obvious problems including violation of the protocol; (S) poor signal strength defined as <15 dB; (C) wrong centration of scan; (A) algorithm failure; (R) retinal pathology other than MS related; (I) illumination; and (B) beam placement]. The fifth criterion describes the any form of retinal pathology (R) which may influence the OCT data. Because the list of diseases in this category is likely to grow further with increasing use of OCT we have summarised our current consensus in Table 2.

**Table 1:** The OSCAR-IB quality control criteria for retinal OCT scans.

Item	criteria
O	Obvious problems not covered by items below.Please document for discussion+consensus agreement
S	Is the OCT signal sufficient?Signal strength >15 (ring and volume scans) with appropriate averaging of multiple scans (ART activated).
C	Is the ring scan correctly centred?for circular discs: ONH must not cross more than two colours of the RAF logo (outer ring of RAF adjusted to outer ring of scan either by paper or electronically). In contrast to the ONH ring scan, post-hoc readjustment is possible for the macular volume scan.
A	Is there an algorithm failure?Red lines correctly identify the superior and inferior RNFL border (ring scan); Red lines correctly identify the retinal borders (voumen scan)
R	Is there visible retinal pathology which may potentially impair the RNFL reading?See Table 2 (note these some of these conditions are also exclusion criteria for OCT studies in MS)
I	Is the fundus well illuminated?Retinal structures visible (ring and volume scans)
B	Is the measurement beam placed centrally?Homogeneous outer ONL reflectivity (ring and volume scans)

**Table 2:** Pathology of the retina to be considered by the OSCAR-IB criteria.

Summary	Diseases
Structural	Drusen, Cysts, Detachment, Large discs, Small crowed discs, Presence of myelinated axons, naevus, tumor, peri-papillary atrophy, optic disc oedema, more than 6 diopters of myopia or hyperopia.
Vascular	AION & PION, NA-AION & NA-PION, GCA, CRO, CRBO, AVM, Cotton-wool spots, CVA affecting the optic pathways
Immune	paraneoplastic, MAR, NMO, CAR, SLE, uveitis, birdshot retinochoroiditis
Infectious	viral, bacterial, fungus, HIV, Lyme, Secondary syphilis
Hereditary	Leber's, DOA, Albinism, Cone dystrophy, Retinitis pigmentosa
Iatrogen	Retina surgery, photocoalgulation, Solar retinopathy, Central serous chorioretinopathy, Purtscher's retinopathy, optic nerve sheet fenestration, Brain surgery affecting the optic pathways
Metabolic/toxic	diabetes, Vit A deficit, Alcohol-, tobacco- and malnutrition-induced amblyopia, Amiodarone, Chloroquine, Vigabatrin
Other	Glaucoma, Macular degeneration, Acute posterior multifocal placoid pigment epitheliopathy, Acute macular neuroretinopathy

Optic disc oedema in MS type optic neuritis (MSON) typically resolves within 1–2 months such that the first signs of RNFL loss following MSON can occasionally be observed after 2 months. For a literature review it is recommended to leave a 3 months time-frame before including RNFL data from these patients into analyses of RNFL loss. [16].

Using the seven new “OSCAR IB” criteria improved the inter–rater agreement from moderate to substantial (kappa 0·61) for re-assessing the same set of OCT scans from Hamburg. The comparison between the first rating and the second rating based on the “OSCAR IB” criteria is shown in Table 3. Note that the highest rejection rate was based on the new criteria on placement of the measurement beam (B).

**Table 3:** Comparison of published criteria with the “OSCAR IB” criteria.

Criterium	Rater 1	Rater 2	Rater 3
Decentration	8/101 (8%)	3/101 (3%)	11/101 (11%)
Algorithm failure	0/101 (0%)	1/101 (1%)	5/101 (5%)
Image quality	13/101 (13%)	5/101 (5%)	8/101 (8%)
Total	19/101 (19%)	9/101 (9%)	17/101 (17%)
O	2/101 (2%)	6/101 (6%)	1/101 (1%)
S	5/101 (5%)	7/101 (7%)	4/101 (4%)
C	7/101 (7%)	5/101 (5%)	5/101 (5%)
A	2/101 (2%)	1/101 (1%)	3/101 (3%)
R	0/101 (0%)	0/101 (0%)	0/101 (0%)
I	4/101 (4%)	6/101 (6%)	10/101 (10%)
B	51/101(51%)	35/101 (35%)	37/101 (37%)
Total	70/101 (70%)	60/101 (60%)	60/101 (60%)

The proportion of *rejected* OCT scans per reader for each of the published and new OSCAR IB criteria is shown for the training set of 101 OCT scans from Hamburg.

The inter–rater agreement remained substantial (kappa 0.61) for external validation, rating an independent set of OCT scans (n = 159) applying the OSCAR-IB criteria.

The total number of rejected OCT scans from the pooled prospective validation set was high (42%–43%) in each of the readers (Table 3). The proportion of rejected OCT scans for each of the “OSCAR IB” criteria showed that the rejected scans frequently failed on more than one single criterion. For this reason there was some variation over the main criterion of rejection documented by the readers (Table 3). An almost perfect agreement was achieved in judging de-centration artifacts (Table 3). As with the training set, the highest disagreement between readers was found for beam placement (B), with reader one preferring to label a scan as “B” when readers two and three labeled them as “I”, “S” or “O”. Of note, in the two data sets there was a very low rate of scans showing retinal pathology other than MS related (n = 2). In one patient this was due to peripapillary atrophy and in the other due to serous retinopathy.

## Discussion

OCT is a new imaging biomarker allowing for rapid, non-invasive and highly precise quantification of axonal degeneration and neuronal loss in the retina of patients suffering from MS [1]–[5]. If successful, retinal OCT may become a key secondary outcome measure in MS treatment trials. Importantly there are to date no consensus quality control criteria to this purpose. This study identified pitfalls which can be reliably identified by trained OCT readers. These pitfalls may not necessarily be apparent to all MS treating neurologists. The unexpected high rejection rate of 42% of retinal OCT scans from experienced centres participating in this study highlights the need for such quality control criteria.

The here presented novel, consensus quality control criteria, OSCAR-IB achieved a high inter-rater agreement (substantial, kappa 0.61). There are to the best of our knowledge no previously published data on inter-rater agreement in this context. Using experience from patients with macular degeneration or glaucoma [6] on our dataset of patients with MS was not convincing (kappa 0.45). The OSCAR-IB criteria allow rating of OCT images in a stringent and systematic approach. In this study the criteria were applied to ringscans of the optic nerve head, which provides an accurate and reproducible measure for RNFL loss in MS by many groups world-wide [2].

**Figure 1:**
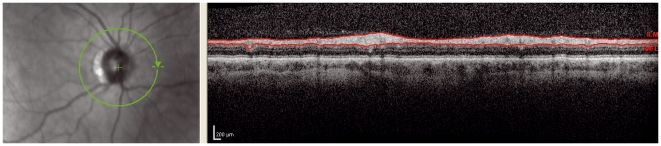
Obvious: The left image is blurred due to poor focusing. This results in increased noise and loss of transversal resolution in the OCT image on the right.

The first of the OSCAR-IB criteria permits to rate and exclude scans on the basis of obvious failures which, if seen frequent, may be included more specifically in a future revision of these OCT quality control criteria (Figure 1). From our own anecdotal observations and the ophthalmological literature we anticipate that large floaters causing shadowing of the OCT image may be an obvious rejection criterium. Equally any damage to the optical pathway from corneal scars, severe keratitis sicca, lens opacities, vitreous haemorraghe to name but few are likely to preclude acquisition of a well illuminated and evenly focused retinal OCT image. In a number of scans rejection was due to more than one of the seven OSCAR-IB criteria. The reason for rejection requires further discussion. One obvious reason was violation of the protocol (no B-scan averaging (ART) enabled in eight scans), discovered by rater three. These eight scans were also very poorly illuminated, taken with off-center beam placement or had a poor signal strength (raters one and two). The influence of ART on image quality in addition to signal strength may need to be considered in a future revision of the criteria.

**Figure 2:**
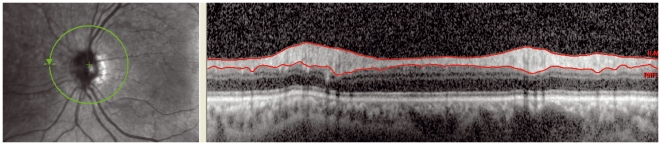
Signal: The signal strength for this image is 13 dB which is lower than the limit of 15 dB. This results in a more noisy OCT image with a lot of speckling.

This is further illustrated by analysing the scan judgement based on the second criterion alone. We had arbitrarily defined that the signal strength had to be larger than 15 dB. There is published evidence that differences in signal strength are associated with differences in average RNFL thickness [9], [10]. Very recently Huang and colleagues showed a signal strength below 7 dB to be negatively correlated with RNFL thickness [11]. For the Heidelberg Spectralis there is however no systematic study investigating the combined influence of signal strength and number of averaged scans needed to achieve reliable performance of the automated algorithm for determination of RNFL thickness. Such studies are required for future refinement of this criterion. Probably the overall contrast between different retinal layers in the final image will become more relevant than one simple measure of signal strength. The rational for setting the limit arbitrarily to a threshold of 15 dB was that we learned from the training set data signal strength was sufficient to prevent algorithm failures due to noise (Figure 2). Based on this proposed cut off of 15 dB only 3–5% of scans were excluded per rater due to poor signal strength.

**Figure 3:**
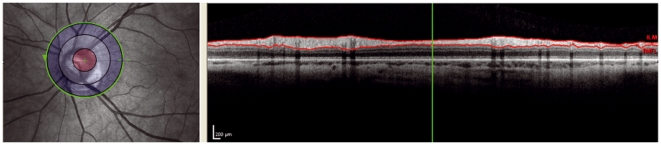
Decentration: The ring scan is not correctly centred as can be observed in the left image. The edge of the optic nerve head crosses more than two circles. Therefore the ringscan is rejected.

The third of the OSCAR-IB criteria, (‘C’) addresses de-centration artefacts. De-centration was found to be one of the main sources of disagreement in the first exploratory part of this study. Overall, 22 scans were rejected by at least one rater due to de-centration and a complete agreement of all three raters was only achieved for two scans. This poor inter-rater agreement is in accordance with the ophthalmological literature [6]. An important hurdle here was the large anatomical variation of the ONH between patients. Allowing for variation in size we argued that a small degree of de-centration of the ring scan around a small ONH would be acceptable because all retinal axons were sampled were they were already spread out in bundles. In contrast, a small degree of de-centration for a large ONH was considered to introduce a significant measurement bias if one part of the ring scan cut through the border of the disc where retinal axons merged and the other part more peripherally. To allow addressing this variation we used a circle with inner rings set at different diameters, a RAF logo (Figure 3). In cases, where the ONH crosses more than two colour bands of the RAF logo, a scan is rejected. Applying the third OSCAR-IB criterion substantially improved the overall agreement on judging de-centration artefacts. The number of OCT scans rejected on the basis of this criterion was about 5% and comparable between raters (Table 3). This finding is consistent with the ophthalmological literature where de-centration artefacts by trained raters occurred in about 5% of the OCT scans sent out to a central reading center [6]. Hopefully, in the near future de-centration artefacts will be recognized by raster scan protocols that identify the center point [6]. Until then, utilization of the RAF logo seems reasonable.

**Figure 4:**
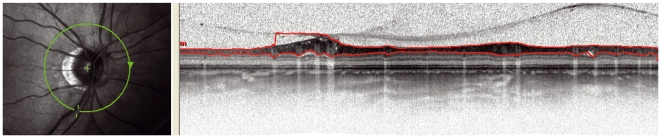
Algorithm failure: The red line in the OCT image right is not clearly at the border of the RNFL. The location corresponds to inferior of the ONH.

The fourth criterium (‘A’) takes boundary line errors or algorithm failures into account. Although these are frequently occurring errors in ophthalmological diseases (15.3%) [6], they only occurred in about 6% in our study (Figure 4). The most likely explanation is that algorithm failures are more frequent in cases with a very thin RNFL, which is rarely the case in MS patients.

**Figure 5:**
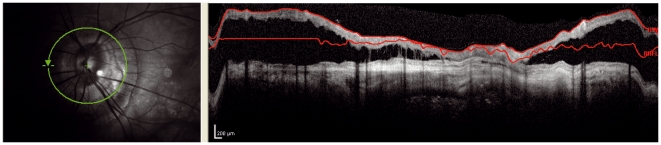
Retinal pathology: There is severe peri-papillary atrophy. It can be seen that this affects the RNFL enormously.

The fifth criterium (‘R’) is retinal pathology not caused by MS (see Table 2). Not only could other retinal pathology generally impair reading of the RNFL (Figure 5), but some diseases such as glaucoma cause RNFL thinning independently of MS and should thus be taken into account [12]. This list is likely to grow as OCT will be used for more diseases across all medical specialities. We propose to keep “R” as a separate criterion in a future revision and regularly amend the list of diseases summarised in Table 2. Such future studies should also carefully revise whether or not severe myopia or hyperopia is a hard criterium for rejection or if these patients could be used as their own controls in longitudinal studies.

**Figure 6:**
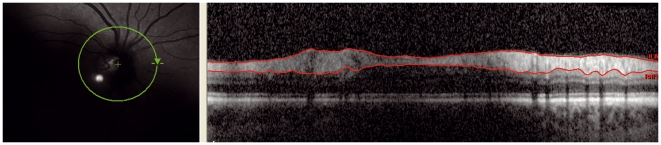
Illumination: The OCT scan here is badly illuminated. Also here this results in speckling and decrease of resolution.

One of the main sources of disagreement in the OCT training set was scan quality. In fact most scans from the training set were rejected due to poor illumination (Figure 6). Therefore illumination has been put forward as the sixth criterium (‘I’).

**Figure 7:**
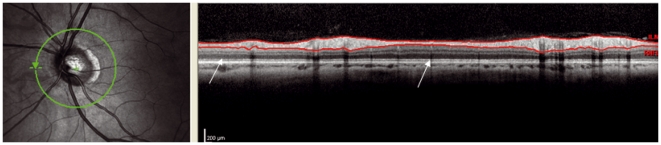
Beam placement: the laser beam is not placed centrally. This can be seen at the outer nuclear layer (ONL). The two arrows point to two regions of the ONL. The left arrow points to a light gray region whereas the other points to a darker gray region. If there is too much difference in colour of the ONL itself a scan is rejected.

The last criterium concerns beam placement (‘B’). Recent literature has demonstrated that off-center beam placement causes an RNFL artifact in a range of up to 10 µm, which by far exceeds annual loss of 1–2 µm RNFL thought to be due to neurodegeneration in patients with MS [13], [14]. This is a new finding which was not known to any of the participating centers at time of OCT scan acquisition. Not surprisingly a large number of scans were rejected based on this criterion alone. Re-applying this criterium to the training set resulted in a 60–70% rejection rate which seems excessive (Table 3). A more realistic but still high (16–28%) rejection rate for the “B” criterium was seen in the validation set (Table 4). Whilst taking an OCT scan it is easy to place the laser beam off-center. Off-center beam placement causes the live image to tilt. The tilting depends on the direction of beam misplacement [14]. Especially in multi-center studies this criterion is of utmost importance since in contrast to live images tilting during scan acquisition is not necessarily visible on averaged summary scans transferred to a central reading center. However, these artefacts can be detected even in averaged summary scans by looking at the outer nuclear layer (ONL) to the ONL reflectivity (Figure 7) [14]. The rigorous application of the “B” criterium was considered necessary because of the large error (up to 42 µm) introduced into measurement of the RNFL thickness. The resulting high rejection rate of almost a third of all scans may come as a disappointment, but hopefully will contribute to improving future retinal OCT studies in MS where an annual RNFL thinning of 2 µm is anticipated [15].

**Table 4:** Proportion of *rejected* OCT scans per reader, broken up to each of the seven “OSCAR IB” criteria based on the prospective validation set of 159 OCT scans from Amsterdam, San Francisco and Calgary.

Criterium	Rater 1	Rater 2	Rater 3
O	0/159 (0%)	4/159 (3%)	8/159 (5%)
S	8/159 (5%)	7/159 (4%)	5/159 (3%)
C	11/159 (7%)	11/159 (7%)	11/159 (7%)
A	10/159 (6%)	10/159 (6%)	12/159 (8%)
R	2/159 (1%)	1/159 (1%)	1/159 (1%)
I	19/159 (12%)	18/149 (1%)	34/159 (21%)
B	45/159 (28%)	29/159 (18%)	25/159 (16%)
Total	67/159 (42%)	68/159 (43%)	67/159 (42%)

There are some limitations about our study. A possible drawback of the “OSCAR-IB” criteria could be that rating in such a way could be too strict, leading to a high rejection rate of OCT scans. In addition, the proposed criteria were applied to ring scans only, but are expected to be also applicable to volume- and other scans as well. Further studies have to be carried out to verify this. In fact, given the increasing evidence for damage to the macula in patients with multiple sclerosis such studies are warranted. From the opththalmological literature one would expect an increased rejection rate based on algorithm failure either due to a very thin RNFL or other structural problems such as for example an epiretinal membrane [6]. Whether such scans would also need to be rejected or require hand-correction of the automated algorithm we are unable to tell from the present study. Another weakness of our study is that the criteria were not validated for retinal OCT images obtained from machines produced by other manufacturers. One would expect similar artefacts and image acquisition problems in OCT machines from other manufacturers, but is difficult to indicate to what extent. For algorithm failures and signal strength one could expect a difference in rejection rate between machines since these artefacts are entirely machine and type of algorithm dependent. On the other hand the rejection rate associated with other criteria are partially influenced by human subjects or raters, thus less machine dependent. Further studies are required to investigate this.

We believe that one of the requirements of MS OCT criteria is that their application should remain simple and practical in daily routine. In the future new findings and new parameters should of course be taken into consideration if these lead to a higher detection rate of artefacts. To conclude, the OSCAR-IB criteria are a first step towards a consensus on quality assessments of retinal OCT measurements in MS. We propose these criteria to be considered for future OCT studies in MS research and to be regularly revised as our experience with this new technology grows.
